# Evaluation of Pollen Apps Forecasts: The Need for Quality Control in an eHealth Service

**DOI:** 10.2196/jmir.7426

**Published:** 2017-05-08

**Authors:** Katharina Bastl, Uwe Berger, Maximilian Kmenta

**Affiliations:** ^1^ Research group Aerobiology and pollen information Department of Oto-Rhino-Laryngology Medical University of Vienna Wien Austria; ^2^ Research group Paleobotany Department of Paleontology University of Vienna Wien Austria

**Keywords:** pollen forecast, pollen information service, mobile app, pollen forecast quality, allergen avoidance

## Abstract

**Background:**

Pollen forecasts are highly valuable for allergen avoidance and thus raising the quality of life of persons concerned by pollen allergies. They are considered as valuable free services for the public. Careful scientific evaluation of pollen forecasts in terms of accurateness and reliability has not been available till date.

**Objective:**

The aim of this study was to analyze 9 mobile apps, which deliver pollen information and pollen forecasts, with a focus on their accurateness regarding the prediction of the pollen load in the grass pollen season 2016 to assess their usefulness for pollen allergy sufferers.

**Methods:**

The following number of apps was evaluated for each location: 3 apps for Vienna (Austria), 4 apps for Berlin (Germany), and 1 app each for Basel (Switzerland) and London (United Kingdom). All mobile apps were freely available. Today’s grass pollen forecast was compared throughout the defined grass pollen season at each respective location with measured grass pollen concentrations. Hit rates were calculated for the exact performance and for a tolerance in a range of ±2 and ±4 pollen per cubic meter.

**Results:**

In general, for most apps, hit rates score around 50% (6 apps). It was found that 1 app showed better results, whereas 3 apps performed less well. Hit rates increased when calculated with tolerances for most apps. In contrast, the forecast for the “readiness to flower” for grasses was performed at a sufficiently accurate level, although only two apps provided such a forecast. The last of those forecasts coincided with the first moderate grass pollen load on the predicted day or 3 days after and performed even from about a month before well within the range of 3 days. Advertisement was present in 3 of the 9 analyzed apps, whereas an imprint mentioning institutions with experience in pollen forecasting was present in only three other apps.

**Conclusions:**

The quality of pollen forecasts is in need of improvement, and quality control for pollen forecasts is recommended to avoid potential harm to pollen allergy sufferers due to inadequate forecasts. The inclusion of information on reliability of provided forecasts and a similar handling regarding probabilistic weather forecasts should be considered.

## Introduction

### Background

Pollen allergies are a global health concern [1]. They affect a considerable fraction of the population in various countries to a considerable extent [2-5]. Pollen forecasts have proven to be a highly valuable tool for allergen avoidance, handling, and treating pollen allergies [6,7]. Pollen information is consumed more often during the pollen season [8]—an indication regarding the need for such services. Today pollen forecasts are also distributed via mobile technology (mobile phones, tablets, any wireless devices) and mobile health (mHealth) has gained importance in terms of informing the public. However, eHealth is still in need for developing standards and the ethical framework [9] to avoid unintended consequences [10]. Sawand et al [11] provided a recent review on secure eHealth monitoring. In addition, a global survey on eHealth was performed by the World Health Organization [12] which reported an increase in eHealth or mHealth activities in countries with higher incomes.

Data quality is a concern [13] and can be judged by various criteria such as accuracy, precision, completeness, timeliness, relevance, legibility, accessibility, usefulness, currency or freshness, and confidentiality. The quality of data or information given in any eHealth service especially in terms of accuracy, precision, and completeness is not always examined; among them mobile apps for food composition [14], diabetes [15], complex chronic disease and disability [16], iron intake [17], lupus management [18], or an online portal for patients with dementia [19] are good examples. All these studies indicate a need for an improvement in quality and usability in eHealth or mHealth services.

### Pollen Forecasts

A range of mobile apps relevant to pollen allergies which provide pollen forecasts are freely available, but users have not been informed about the accurateness of pollen forecasts till date. Simultaneously, the identity of the publishers of such apps with health-related content for pollen allergy sufferers has not been available. Also, studies on apps with a focus on pollen allergies and allergen prevention are still rare [20,21]. Five of the apps examined herein were included in a study by Berger et al [20] evaluated concerning their functionality and content. The criteria in the Berger et al study were different from this study and examined the app from a formal point of view, but did not consider the quality of the pollen forecast itself. Although parameters of good scientific practice for pollen forecasting were recently defined [22], it is not known how different apps perform concerning their pollen forecast quality. Questions regarding the extent to which pollen forecasts represent reality and how forecasts perform concerning their prediction of the readiness to flower remained unanswered.

Herein, we analyze the grass pollen forecasts of 9 mobile apps from four different countries during the grass pollen season of 2016. Moreover, the forecast dates for the readiness to flower for grasses in order to examine the accurateness and reliability of these forecasts was evaluated. Gaps and errors in the forecasts are also considered in order to complete the picture of the performance of specific apps.

## Methods

### Selection of Mobile Apps Included

The following free downloadable apps were included in this study: (1) “Pollen” [23], (2) “Biowetter,” (3) “Pollenwarner,” (4) “DWD Pollenflug-Index” (short: “DWD”), (5) “Allergiehelfer,” (6) “Pollenflug Vorhersage” (short: “Pollenflug”), (7) “Allergohelp Deutschland” (short: “Allergohelp”), (8) “Pollen News,” and (9) “Hayfever pollen forecast” (short: “Hayfever”). Apps 1-3 were used for Austria and their forecast for Vienna was evaluated. Apps 4-7 were used for Germany and their forecast for Berlin was evaluated. App 8 was used for Switzerland and its forecast for Basel was evaluated. App 9 was used for the United Kingdom and its forecast for London was evaluated. Every day a screenshot of each app forecast was made at the same time (1 PM) during the study period, so that always an up-to-date forecast (the day’s forecast) was used for the evaluation. Forecasts were not analyzed 2 or 3 days ahead in order to examine the base accurateness of the pollen forecasts. The forecasted load for grass pollen (Poaceae) was analyzed for the evaluation of the forecasts, since the grass pollen season is a long pollen season and a less fluctuating one, thus one of the more simple ones to forecast.

### Datasets

The pollen data used was retrieved from the European Aeroallergen Network (EAN) database and originates from the national pollen monitoring networks (see “Acknowledgments”). Pollen data was attained by Hirst-type pollen traps [24]. Operation of those volumetric traps as well as the light microscopic evaluation of the resulting air samples follow the minimum requirements of the European aerobiology community [25] also required from the EAN database assuring high quality of the pollen data. The grass pollen season was defined on the EAN standard definition from 1% to 95% of the annual grass pollen index for each location based on local grass pollen data: Vienna (May 1, 2016 to August 5, 2016), Berlin (May 12, 2016 to July 23, 2016), Basel (April 30, 2016 to August 8, 2016), and London (May 28, 2016 to July 22, 2016).

### Statistics

Descriptive statistics and R3.3.1 (R Foundation for Statistical Computing, Vienna, Austria) were used for the analysis. Daily grass pollen concentrations were compared within this defined grass pollen season with the forecasted loads. Used load classes and their respective pollen concentration ranges are shown in Table 1. The ranges used are adopted from the Austrian pollen information service and partly correspond to the ranges also used in Germany, Switzerland, and the United Kingdom. A tolerance between the load categories was introduced to allow flexibility around the boundaries (due to biogeographical differences or different pollen load definitions). A correct prediction in this setting means that the predicted load is the “exact” category or the category computed with the increased or decreased value. We define herein “hit rate” as the measure of the pollen forecast performance concerning accuracy of forecasted daily pollen concentrations, that is, the rate of correct forecasts within a defined time period. The calculations comprise an exact hit rate (no tolerance), a hit rate of ±2 pollen, and a hit rate of ±4 pollen around the boundary of each category (Table 2). This is because a forecast should not be falsified automatically due to a small difference in overlap (eg, of one pollen grain). An adopted approach had to be developed for the “DWD” app since it also uses intermediate forecast loads in contrast to all other apps which should overlap with two neighboring categories. Numerical intervals were computed based on the mean between the load boundaries and a 20% interval (see Table 1). For the exact match, the ratio of correct predictions was computed (in the case of the “DWD” app, if the pollen concentration was in the overlapping interval, both categories was counted as correct).

In addition, the performance of the forecast “readiness to flower” for grasses was compared with the first-occurring moderate pollen load in the season. The first moderate grass pollen load was chosen as “threshold,” as low grass pollen loads can occur in the air preceding the grass pollen season. However, only two pollen apps (“Pollen” and “Pollen News”) provided such a service.

## Results

### Description of the Mobile Apps

The mobile apps under study herein are characterized concerning their main options, functional background, and noteworthy observations. All of them are available for free.

“Pollen” comprises pollen information (forecasts, countdown for the season, different pollen dispersal models, and forecast maps), services concerning pollen allergy (diary for allergic symptoms and burden, daily load based on symptom entries, personalized pollen information, search for allergologists), botanical information, push notification services, and an imprint (the Austrian pollen information service is the developer and operator). To the best of our knowledge, it is the only app described and published scientifically [23] among the apps studied herein.

**Figure 1:**
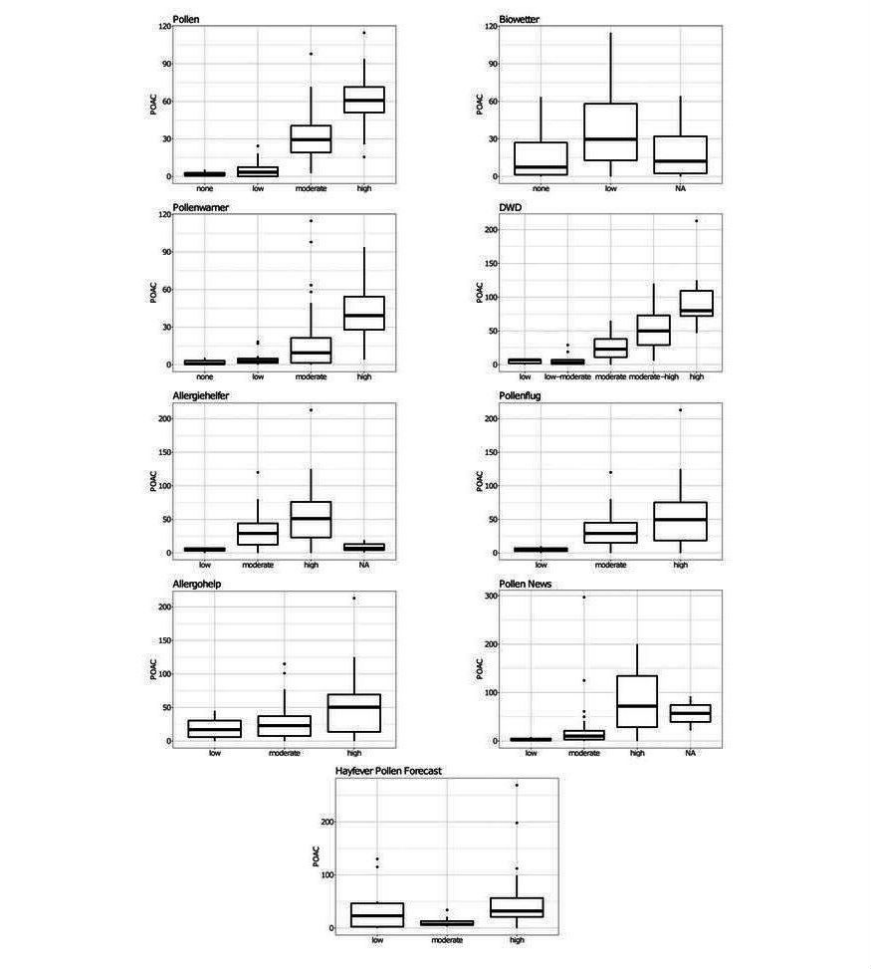
Boxplots of the apps under study and their performance concerning forecasting grass pollen loads (none, low, moderate, high) and missing forecasts (NA).

“Biowetter” is an app focusing on weather and biorhythm, thus pollen information is only one part of the app. An imprint does not exist and advertisement appears regularly in the app. During the study period, it was the app with the most frequent crash reports and missing forecast information (Figure 1).

“Pollenwarner” includes pollen information, push notification services, a symptom diary, and general information (tips and tricks). An imprint is not available, but viewing the logos indicates that the companies Tempo and Otriven are connected with this app.

**Table 1:** The forecasted load classification is shown along with the respective pollen concentrations used in this study. The intermediate stages (no-low, low-moderate, moderate-high) are used only by one app (“DWD”).

Forecast load	Daily pollen concentrations
No	0-0.99
No-low	0-2
Low	1-19.9
Low-moderate	15.6-23.4
Moderate	20-49.9
Moderate-high	39.6-59.4
High	50 and above

**Table 2:** The results of the hit rates are shown per app and per analyses (ie, exact hit rates and hit rates with tolerances of ±2 or ±4 pollen, respectively). Note the increasing hit rates in some apps (such as “Pollen,” “Allergiehelfer,” and “Pollenflug”) versus the less improving apps (such as “Hayfever”).

Mobile apps	Exact hit rates (%)	Hit rates with tolerance 2 (%)	Hit rates with tolerance 4 (%)
“Pollen”	62.9	77.3	80.4
“Biowetter”	31.8	39.4	42.2
“Pollenwarner”	34.0	39.2	42.3
“DWD”	41.1	46.6	52.1
“Allergiehelfer”	48.6	54.3	57.1
“Pollenflug”	50.7	56.2	58.9
“Allergohelp”	45.2	50.7	52.1
“Pollen News”	42.4	48.5	51.5
“Hayfever”	35.7	39.3	39.3

“DWD” provides pollen forecasts, a forecast map, and possesses an imprint, “Deutscher Wetterdienst,” which is not only the official German weather service, but also a higher federal authority in the business area of the Federal Ministry of Transport and Digital Infrastructure that provides pollen forecasts based on the pollen measurements from the Foundation German Pollen Information (PID).

“Allergiehelfer” comprises pollen forecasts including push-service and general information. The pharmaceutical company GlaxoSmithKline GmbH & Co. KG is indicated in the imprint.

“Pollenflug” delivers pollen forecasts, a forecast map, push notification services, an allergy questionnaire, and general information. The pharmaceutical company Hexal AG is mentioned in the imprint and the app includes advertisement of allergy medication.

“Allergohelp” provides pollen forecasts, forecast maps, general information on allergy and therapy, therapy documentation, and a search for allergologists. The pharmaceutical company Allergopharma GmbH & Co. KG is mentioned in the imprint.

“Pollen-News” comprises pollen information (pollen forecasts, flowering start, maps) and general information (tips and tricks, information on aeroallergens). The aha! Allergiezentrum Schweiz and Bundesamt für Meteorologie und Klimatologie MeteoSchweiz are indicated as publisher in the imprint. The aha! Allergiezentrum Schweiz sees itself as patient organization, whereas the Bundesamt für Meteorologie und Klimatologie of MeteoSchweiz is the national weather and climate service in Switzerland.

“Hayfever” provides pollen forecasts and forecast maps. Advertisement is present and an imprint does not exist. However, the company A.Vogel (natural and herbal remedies) is present in the form of a logo.

### Results of the Pollen Forecast Quality as Assessed by Hit Rates

The quality of the grass pollen forecast, measured by hit rates (Table 2) varies per app. Considering the exact hit rates, 5 apps attained a score of 40-50% (“DWD,” “Allergiehelfer,” “Pollenflug,” “Allergohelp,” and “Pollen News”), whereas 3 apps attained a lower score, below 40% (“Biowetter,” “Pollenwarner,” and “Hayfever”) and only one app scored above 60% (“Pollen”). The hit rates improved when a tolerance range was calculated and most apps benefitted from an increasing tolerance. Only “Hayfever” showed a low improvement followed by stagnation from a tolerance from ±2 to ±4 pollen. A significant increase in exact hit rates to a tolerance of ±2 was observed in most apps, especially in “Pollen” which showed an increase of about 15%. “Pollen” is also the app with the highest hit rates in total (up to 80% with a tolerance of ±4) and the greatest improvement and thus the smallest error. The next best performances ranged from 50% to 59% (“Pollenflug,” “Allergiehelfer,” “DWD,” “Allergohelp,” and “Pollen News”). Even with the highest tolerance, there were apps with a score below 50% (“Biowetter,” “Pollenwarner,” and “Hayfever”).

Boxplots of the forecasted pollen level for all apps analyzed revealed further insights (Figure 1). Apps with gaps or errors during the grass pollen season were “Biowetter,” “Allergiehelfer,” and “Pollen News.” It is remarkable that “Biowetter” not only showed the highest frequency of missing forecasts, but also forecasted no or a low grass pollen load in contrast to all other apps. Low quality also became evident in a similar distribution of the mean of the forecasted load and the 25-75% quantiles (eg, “Biowetter,” “Allergohelp,” and “Hayfever”).

Grasses were ready to flower based on the forecast countdown of “Pollen” in Vienna on May 9, 2016; and the first moderate grass pollen load was also occurred on the very same day. Grasses were ready to flower based on the forecast countdown of “Pollen News” in Basel on May 10, 2016; and the first moderate grass pollen load occurred 3 days earlier (ie, on May 7, 2016).

## Discussion

### Principal Findings

Forecasting pollen concentrations or loads is not a trivial concern since many factors play a role in the development of the burden for pollen allergy sufferers. Several data sources and information are required including the biogeography of the region, continuous pollen monitoring and reliable pollen data, high quality weather forecasts, phenology, models (numerical simulations of pollen dispersal), medical-allergological expertise respective symptom data, and experience in the task of pollen forecasting [22]. The majority of apps in this study achieved only hit rates of 40-60% (8 apps) for pollen forecasts for the actual day when applying a tolerance range of ±4 pollen. The results of this study indicate that a certain forecast quality is already existent with room for improvement.

Although only two apps provided a forecast on the readiness to flower (“Pollen” and “Pollen News”), those forecasts had an excellent performance. The forecasted dates used here were the ones used when the information “ready to flower” occurred. Earlier forecast dates from mid-April (eg, April 15, 2016) announced for both apps May 6, 2016 as date for grasses ready to flower, which is still accurate enough considering it was a forecast from nearly a month before the start of the respective grass pollen season. The forecasted date coincided with the first moderate grass pollen load (“Pollen”) or within a few days (“Pollen News”) and may thus be recommended as a useful service for pollen allergy sufferers to prepare them for the grass pollen season.

### Limitations

Certain facts and dependencies complicating the generation of an accurate pollen forecast have to be mentioned. Pollen forecasting activities depend on weather forecasts that are as accurate as possible. Although weather forecasting accuracy hit rates range from 85% to 95% for minimum and maximum temperatures on the same day, the forecasts of precipitation and sun hours are more complex and achieve lower rates [26]. Also, forecasts for environmental pollutants such as ozone are dependent on the accurateness of meteorological parameters. For example, the accuracy of ozone forecasts deviated from 6% to 25% in a 3-year evaluation due to meteorological forecast errors [27]. In addition, a consideration should be if pollen forecasts should be treated similarly to probabilistic weather forecasts. Hereby, we want to formulate the question if the scientific community and pollen information services should treat a forecast of a “low pollen load” as “high probability of a low pollen load” with all consequences ranging from defining probabilities and promoting and communicating it in an understandable way to the public which is already difficult for probabilistic weather forecasts [28]. Also, pollination varies with plant abundance and microclimate resulting in different pollen concentrations in the same locality or city [29-31]. This fact was considered in this study regarding the analyses of hit rates with tolerances. We propose that analyses with tolerances, such as used herein, are advantageous because they compensate for minor discrepancies that might not be attributed to as errors. Missing pollen forecasts are a problem for some apps (4 apps) and a problem of apps published by companies. There is a need for improvement in reliability of the service since no information at all is as useless as inaccurate pollen forecasts for allergy sufferers concerned. It is obvious that a quality control of forecasts would be a benefit for pollen allergy sufferers to inform them about the reliability of the pollen forecasts of specific suppliers and to improve the quality of pollen forecasts.

### Recommendations and Possible Gateways for the Future

Mobile apps dealing with allergen avoidance with the aim of supporting pollen allergy sufferers should fulfill certain criteria and functionalities, among them easily understandable pollen forecasts, a minimum of forecasted aeroallergens, botanical information, symptom diaries, allergy risk questionnaires, and an imprint with the publisher of the app stating the responsible institution, at best without conflict of interests [20]. Herein, it should be noted that advertisement is common in pollen information apps, especially if the publisher is a company and not directly involved in pollen forecasting. Apps with direct advertisement are “Biowetter,” “Pollenflug,” and “Hayfever.” Who the publisher of an app actually is represents crucial information. An institution involved in forecasting is in the background of the apps “Pollen,” “DWD,” and “Pollen News” in terms of occurrence in the imprint, but not in the six other apps. The need for an improvement and quality control of pollen forecasts is underlined by the outcome of this study.

A combination of data sources and methods will lead to an improvement of pollen forecasts and is already used in the mobile app with the best performance found in this study. Phenological routines assess the local progression of the pollen season. Pollen dispersal models and readiness to flower models support the person preparing the pollen forecast. Symptom data reveal the impact of the daily pollen concentrations on pollen allergy sufferers and allows for tailoring the forecast to the needs of persons concerned. These are possible gateways in the need for a tighter connection to a future pollen forecast. Specific recommendations concerning the improvement of pollen forecasts comprise (1) ongoing evaluation of pollen forecast quality at best also during the pollen season to raise quality as fast as possible, (2) comprehension of the probabilistic nature of pollen forecasts and implementation of this aspect in services visible for users (such as percentual information on the forecast accuracy), and (3) implementation of all information sources necessary such as symptom data and phenological routines besides pollen data to improve a pollen forecast [22].

### Conclusions

Pollen forecasts are essential for pollen allergy sufferers in terms of allergen avoidance and thus the accuracy of such forecasts is a key factor for improving the quality of life. Most apps deliver forecasts with a hit rate of about 50%, which is a score that is too low for this purpose. Quality control of pollen forecasts should be introduced since wrong forecasts can be seen as potential physical injury and may harm persons concerned significantly. Pollen information and pollen forecasts should never be given out by pharmaceutical companies or be accompanied by advertisement [22] to ensure unbiased pollen forecasting. Finally, we have determined that proof of knowledge and necessary datasets for institutions developing or promoting apps with pollen forecasts is necessary and vitally important to ensure the safety of users and the quality of this eHealth service.

## Abbreviations

EAN: European Aeroallergen Network
